# Multiple Large Prostatic Stones Causing Chronic Pelvic Pain: A Case Report

**DOI:** 10.7759/cureus.20583

**Published:** 2021-12-21

**Authors:** Ajay Nimbalkar, Vinal More, Sony Mehta

**Affiliations:** 1 General Surgery, Mumbai Port Trust Hospital, Mumbai, IND; 2 Urology, Mumbai Port Trust Hospital, Mumbai, IND

**Keywords:** chronic pelvic pain, lower urinary tract symptoms, bladder diverticula, chronic pelvic pain syndrome, prostate stones

## Abstract

Apart from a few cases, prostatic stones are asymptomatic and found incidentally on routine evaluation. Current knowledge about the significance of prostatic stones in urological symptoms and chronic pelvic pain syndrome is limited. Although prostatic stones are rare, they are frequently present in patients with chronic pelvic pain syndrome and increase inflammation and duration of symptoms in these patients. We report an unusual case of a 70-year-old male who presented with lower urinary tract symptoms and chronic pelvic pain with large multiple prostatic stones and urinary bladder diverticula, which was managed endoscopically.

## Introduction

The incidence of prostatic stones varies from 7% to 70%. They are usually asymptomatic and most cases are found incidentally on transrectal ultrasonography during the evaluation of benign prostatic hyperplasia [1]. In a few cases, they cause severe lower urinary tract symptoms and bladder outlet obstruction. The origin of prostatic stones is still debatable, but a distinction must be made between primary endogenous versus secondary exogenous stones. The incidence of chronic pelvic pain syndrome varies from 2% to 16% and the negative effect on the quality of life is comparable to chronic diseases such as diabetes, myocardial infarction, and inflammatory bowel disease [2]. Prostatic stones are frequently present in patients with chronic pelvic pain syndrome and cause increased prostatic inflammation and duration of symptoms in these patients [3]. In our report, we present an unusual case of multiple large prostatic stones with multiple urinary bladder diverticula causing chronic pelvic pain, which was managed endoscopically.

This case report was previously presented as a poster at the 42nd annual conference of the Maharashtra state chapter of the Association of Surgeons of India (ASI) on January 25, 2020.

## Case presentation

**Figure 1:**
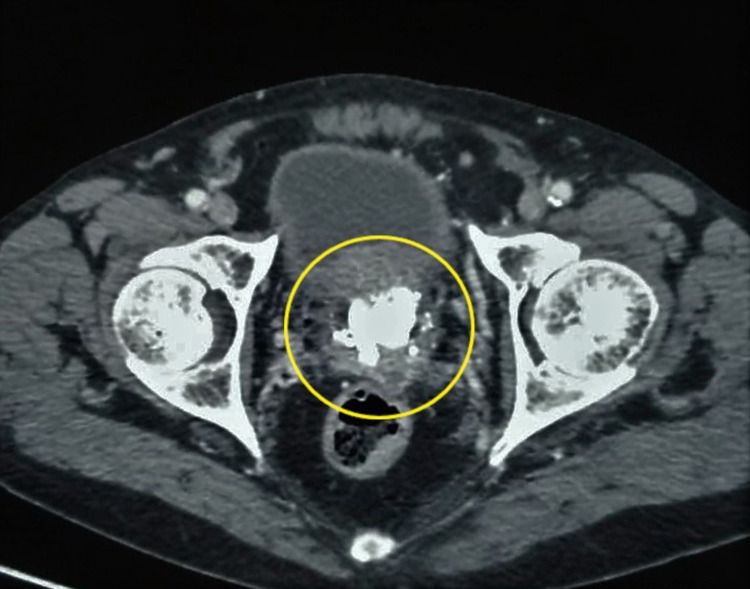
Contrast-enhanced computed tomography (CECT) scan of pelvis shows multiple stones in the prostate gland (yellow circle)

**Figure 2:**
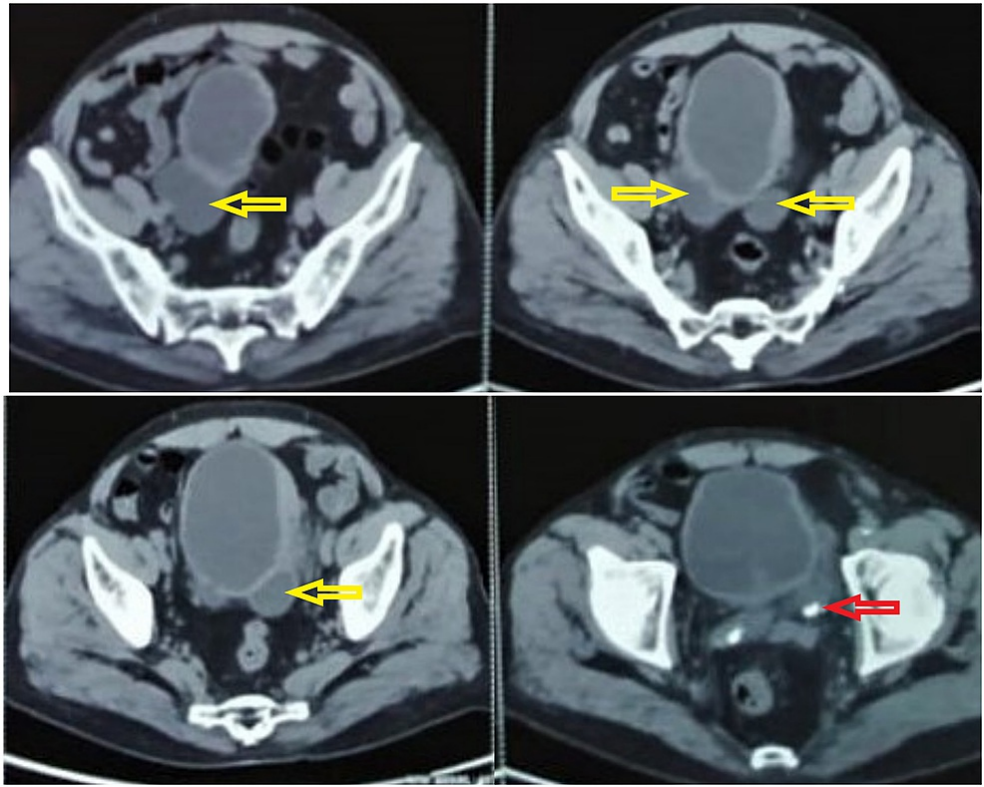
Contrast-enhanced computed tomography (CECT) scan of pelvis shows multiple urinary bladder diverticula (yellow arrows) and stone inside the diverticulum (red arrow)

A 70-year-old male presented with complaints of nocturia, increased frequency of micturition, poor stream of urine, and feeling of incomplete voiding. The patient also complained of a constant dull aching pain in the pelvic region radiating to the back and groin. He had these symptoms for one year, which gradually worsened over the last three months. He had no relief with repeated antibiotics, analgesics, and alpha-blockers that were prescribed to him before he visited our hospital. The patient had no history of fever, burning micturition, cloudy urine, hematuria, or passage of any stone. He had no history of any previous surgery. On digital rectal examination, the patient had moderate prostatomegaly with rolling crepitus. The prostate gland was symmetrically enlarged and hard in consistency. All routine blood investigations were within normal limits. Serum prostate-specific antigen (PSA) was 2 ng/ml. Urine on culture showed no significant growth. Contrast-enhanced computed tomography (CECT) scan of the pelvis was done (Figures 1-2), which was suggestive of multiple stones in the prostate gland and moderate prostatomegaly with multiple diverticula in the urinary bladder. Few stones were present in the urinary bladder diverticula as well.

**Figure 3:**
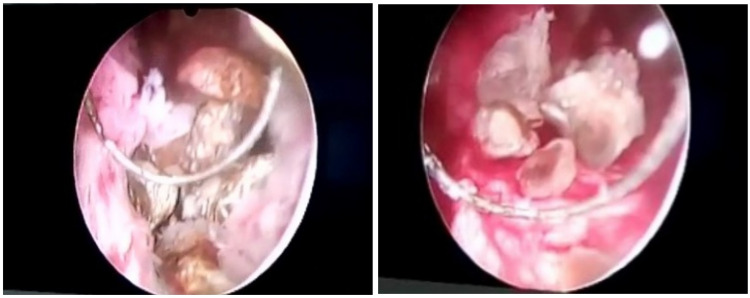
Intra-operative pictures of transurethral resection of the prostate gland showing multiple prostate stones

Uroflowmetry showed a low peak flow rate of 7 ml/sec with significant post-void residue. Transrectal ultrasonography-guided prostate biopsy (12 core) was performed, which was suggestive of benign prostatic hyperplasia with changes of prostatitis. The patient underwent endoscopic removal of bladder diverticula stones and transurethral resection of the prostate with simultaneous removal of prostatic stones in the same setting (Figure 3).

The patient's postoperative recovery was uneventful. The histopathology of prostate bits was suggestive of prostatitis without any evidence of malignancy. Stone composition of both the urinary bladder and prostatic stones on analysis came as calcium phosphate. Postoperatively, the patient was followed up at one week, two weeks, and four weeks. At the fourth week follow-up, the patient showed significant improvement in symptoms with an improved peak flow rate of 17 ml/sec on uroflowmetry.

## Discussion

The exact mechanism of formation of prostatic stones is still debatable. Based on the most accepted mechanisms of prostatic stone formation, they are classified as primary prostatic stones or secondary exogenous stones. Primary or endogenous stones are formed due to increased prostatic secretions, increased corpora amylacea, and blockage of secretory tubes of the prostate gland due to the inflammatory process in the prostate gland. There are more than 30 tubular independent functional units in the prostate gland, which lead to the formation of multiple primary stones. Secondary or exogenous stones are formed as urinary stones. They are formed because of chronic reflux of urine into the prostate, which leads to the deposition of inorganic calcium salts in the prostate gland [4].

Here, the composition of both the prostate and bladder diverticula stones was the same i.e., calcium phosphate. This may indicate a common origin of both stones.

In a few symptomatic cases, prostatic stones present with lower urinary tract symptoms, acute retention of urine, stricture urethra, or chronic pelvic pain [5]. But, contrary to popular belief, few studies have also shown that the presence of prostatic stones was not a significant predictor of moderate or severe lower urinary tract symptoms but increased caliculi burden in the prostate correlated with aggravated storage symptoms [6]. Hence, the exact relationship between prostatic stones and urological symptoms is still not well understood.

The typical symptoms of chronic pelvic pain syndrome are chronic pelvic pain, pain in the urogenital floor, and external genitalia with associated lower urinary tract symptoms [7].

Our patient came with a defining history of chronic pelvic pain syndrome with lower urinary tract symptoms, but the diagnosis of prostatic stones was not made before he visited our hospital. He had no relief with repeated antibiotics, analgesics, and alpha-blockers that were prescribed to him before he visited our hospital. This is usually the case as medical treatment is inadequate and surgical treatment is necessary when the patients are symptomatic of prostatic stones. Unusually, in this case, the patient had simultaneous multiple urinary diverticula with few stones within them suggestive of chronic bladder outlet obstruction due to prostatic stones, which was evaluated with uroflowmetry. The treatment options available for prostate stones were endoscopic transurethral resection of the prostate or open prostatectomy with enucleation of the whole gland [1]. In this case, the patient underwent endoscopic removal of all bladder diverticula stones and transurethral resection of the prostate with simultaneous removal of prostatic stones in the same setting. The patient’s lower urinary tract symptoms and chronic pelvic pain were resolved after the surgery. It is seen that prostatic stones are common in patients with chronic pelvic pain syndrome and they increase inflammation, bacterial colonization, and duration of symptoms in these patients [3]. Though the exact inciting event for chronic pelvic pain syndrome is not known, chronic inflammation is considered a significant factor associated with the interplay between psychological, immune, neurologic, and endocrine factors. This interplay produces the symptoms of chronic pelvic pain syndrome [8]. So it's a combination of these factors along with prostatic stones, which can lead to such a presentation.

## Conclusions

Although prostatic stones are rare, they are frequently present in patients with chronic pelvic pain syndrome and lead to recurrent infections and chronic inflammation, but their significance in the etiology of chronic pelvic pain syndrome is not well understood. Hence, in these types of unusual cases, thorough evaluation is needed for better understanding and management of prostate stones and associated chronic pelvic pain.
